# Increasing the midsole bending stiffness of shoes alters gastrocnemius medialis muscle function during running

**DOI:** 10.1038/s41598-020-80791-3

**Published:** 2021-01-12

**Authors:** Sasa Cigoja, Jared R. Fletcher, Michael Esposito, Darren J. Stefanyshyn, Benno M. Nigg

**Affiliations:** 1 Human Performance Laboratory, Faculty of Kinesiology, University of Calgary, Calgary, AB Canada; 2 Department of Health and Physical Education, Mount Royal University, Calgary, AB Canada; 3 Biomedical Engineering Graduate Program, University of Calgary, Calgary, AB Canada

**Keywords:** Biophysics, Anatomy

## Abstract

In recent years, increasing the midsole bending stiffness (MBS) of running shoes by embedding carbon fibre plates in the midsole resulted in many world records set during long-distance running competitions. Although several theories were introduced to unravel the mechanisms behind these performance benefits, no definitive explanation was provided so far. This study aimed to investigate how the function of the gastrocnemius medialis (GM) muscle and Achilles tendon is altered when running in shoes with increased MBS. Here, we provide the first direct evidence that the amount and velocity of GM muscle fascicle shortening is reduced when running with increased MBS. Compared to control, running in the stiffest condition at 90% of speed at lactate threshold resulted in less muscle fascicle shortening (*p* = 0.006, *d* = 0.87), slower average shortening velocity (*p* = 0.002, *d* = 0.93) and greater estimated Achilles tendon energy return (*p* ≤ 0.001, *d* = 0.96), without a significant change in GM fascicle work (*p* = 0.335, *d* = 0.40) or GM energy cost (*p* = 0.569, *d* = 0.30). The findings of this study suggest that running in stiff shoes allows the ankle plantarflexor muscle–tendon unit to continue to operate on a more favourable position of the muscle’s force–length–velocity relationship by lowering muscle shortening velocity and increasing tendon energy return.

## Introduction

In recent years, world records were set at long-distance running competitions over 5 km, 15 km, half-marathon, marathon, and 100 km distances while running in the Nike Vaporfly 4% or Nike Vaporfly Next% shoes (note: subsequently referred to as ‘Vaporfly’). Several studies investigated the effects of running in the Vaporfly shoes on biomechanical and physiological variables^1–5^. Compared to other footwear, running in the Vaporfly shoes (1) improved running economy by 4% on average^1,4,5^, (2) decreased the negative work and peak extension (i.e., dorsiflexion) angle at the metatarsophalangeal (MTP) joint^2^, (3) decreased the positive and negative work, and peak plantarflexion moment and dorsiflexion angle at the ankle joint^2^, and (4) increased stance times^1,4^. At the time when the long-distance running world records were set, the Vaporfly shoes possessed features that significantly differentiated them from other competitor’s running footwear. The main features included (1) a single curved carbon fibre plate embedded in the midsole, (2) highly resilient midsole material, and (3) a greater stack height (i.e., midsole thickness)^6^. The use of carbon fibre plates to increase the longitudinal midsole bending stiffness (MBS) of shoes was first introduced by Stefanyshyn and Nigg^7^. The MTP joint undergoes substantial extension during the stance phase of running where negative work is performed^8^. This absorbed energy was hypothesised to be disadvantageous for athletic performance^9^. One way to inhibit the extension of the MTP joint and therefore reduce the negative work performed was to increase the MBS of shoes^7^. Stefanyshyn and Fusco^10^ investigated the effects of MBS on sprinting performance and showed that 87% of all participants performed better when they inserted carbon fibre plates in their own shoes. Furthermore, the level of MBS that resulted in best performance differed between sprinters, suggesting that there is a subject-specific optimum in stiffness, which allows athletes to perform at their best. Roy and Stefanyshyn^11^ were the first to investigate if increased MBS can be used to improve long-distance running performance. Their findings showed that the mean rate of oxygen consumption (${\dot{\text{V}}}_{{{\text{O}}_{{2}} }}$) can be lowered when running in a stiffer shoe; however, if MBS was increased too much, ${\dot{\text{V}}}_{{{\text{O}}_{{2}} }}$ rose again, supporting the idea of an optimal level of MBS^10^.

Although the underlaying mechanisms of improved performance when running in footwear with increased MBS are not well understood, multiple effects were proposed: (1) minimising energy loss at the MTP joint^7^, (2) storing and returning elastic energy to the athlete^12,13^, (3) the ‘teeter-totter’ effect^14,15^, and (4) optimising the force–velocity profile of the ankle plantarflexor muscles^16–18^. In brief, the principle of minimising energy loss suggests that athletic improvements can be achieved by reducing the energy absorbed at joints due to less eccentric work performed by muscles crossing these joints. The principle of storing and returning elastic energy suggests that elastic strain energy is stored in carbon fibre plates when being bent as the MTP joint undergoes extension. Due to the plate’s hysteresis, a portion of this stored energy is then speculated to be returned to the athlete during the subsequent flexion of the MTP joint. The ‘teeter-totter’ effect suggests that the ground reaction force (GRF) produces a reaction force at the heel due to the curvature of the carbon fibre plate as the centre of pressure travels anteriorly during the stance phase of running. This heel force was speculated to act at the right location, the right time, and with the right frequency^19^, and therefore potentially improve running economy. The principle of optimising the force–velocity profile of the ankle plantarflexor muscles suggests that the velocity of muscle shortening is reduced due to a change in gear ratio between the external (i.e., distance between GRF and ankle joint centre) and internal (i.e., distance between Achilles tendon (AT) and ankle joint centre) moment arms^20^. Willwacher et al.^16^ demonstrated that, compared to a control shoe, running in stiff shoes increased the average GRF moment arm to all lower limb joint centres. The observed effects were greatest at the ankle joint, the major positive work generator in running^8^. Thus, with a relatively constant internal moment arm, these findings suggest that running in shoes with increased MBS would result in a greater gear ratio. This was speculated to potentially affect the force-generating capacity of the ankle plantarflexor muscles^16,17^. Takahashi et al.^17^ showed that the gear ratio was greater and the shortening velocity of the soleus muscle was reduced when walking in shoes with increased MBS. Interestingly, the metabolic cost was increased when walking in the stiff compared to a control condition. Ray and Takahashi^21^, however, demonstrated that increasing the stiffness of shoes when walking at faster speeds reduced the metabolic cost. This suggested that stiffening elements in shoes had a speed-dependent effect during walking. The fact that metabolic cost increased and altered ankle joint angles were observed in the stiff condition during slow walking is contrary to previously reported findings in running^1,2,11^, and would therefore suggest that different mechanisms occur between these two tasks. For this reason, Cigoja et al.^18^ investigated the effects of MBS on force and velocity of the shank muscle–tendon unit (MTU) (i.e., estimate of triceps surae muscle and AT) during running. Compared to control, it was reported that running in a stiff shoe decreased peak shortening velocities of the shank MTU likely due to increased stance times. The authors speculated that these reduced shortening velocities could have implications on the metabolic cost of running and therefore affect long-distance running performance. Any changes in shank MTU shortening velocities due to the use of carbon fibre plates could result in reduced energy cost of the ankle plantarflexor muscles, and thus whole-body metabolic cost. However, due to the methodologies used in their study, Cigoja et al.^18^ could not address whether the observed differences in shank MTU shortening velocities occurred due to changes at the muscle (i.e., triceps surae) or tendon (i.e., AT).

Thus, the primary purpose of this study was to investigate if the total shortening and the average shortening velocity of the gastrocnemius medialis (GM) muscle is reduced when running in shoes with increased midsole bending stiffness (i.e., Stiff, Stiffer, Stiffest) compared to a control shoe (i.e., Control). It was hypothesised that the total amount of shortening will not differ between footwear conditions because previous studies did not report differences in ankle joint plantarflexion angles^2,11,13,22^. It was further hypothesised that running in footwear with increased MBS will reduce average GM shortening velocities due to increased stance times. The secondary purpose of this study was to investigate if increased midsole bending stiffness of shoes will affect modelled GM energy cost and AT energy return. It was hypothesised that, compared to control, GM energy cost will decrease with increased MBS due to reduced average GM shortening velocities. We further hypothesised that no difference in AT energy return would be observed between footwear conditions because previous studies suggested that primarily the function of muscles would be affected by changes in MBS of shoes^16,17,23^.

## Results

### Energy cost of running

There was no significant effect of MBS on the energy cost of running (E_run_; *p* = 0.298). Overall, two participants had the lowest E_run_ in Control (i.e., Nike Free Run 2018), three in Stiff (i.e., Control + 1 mm carbon fibre plate), seven in Stiffer (i.e., Control + 1.5 mm carbon fibre plate), and two in Stiffest (i.e., Control + 2 mm carbon fibre plate). There was no difference (*p* = 0.25, *d* = 0.13) in E_run_ between running in the first (4.69 ± 0.29 J kg^−1^ m^−1^) and last (4.64 ± 0.25 J kg^−1^ m^−1^) condition.

### Gastrocnemius medialis muscle and Achilles tendon dynamics

**Figure 1:**
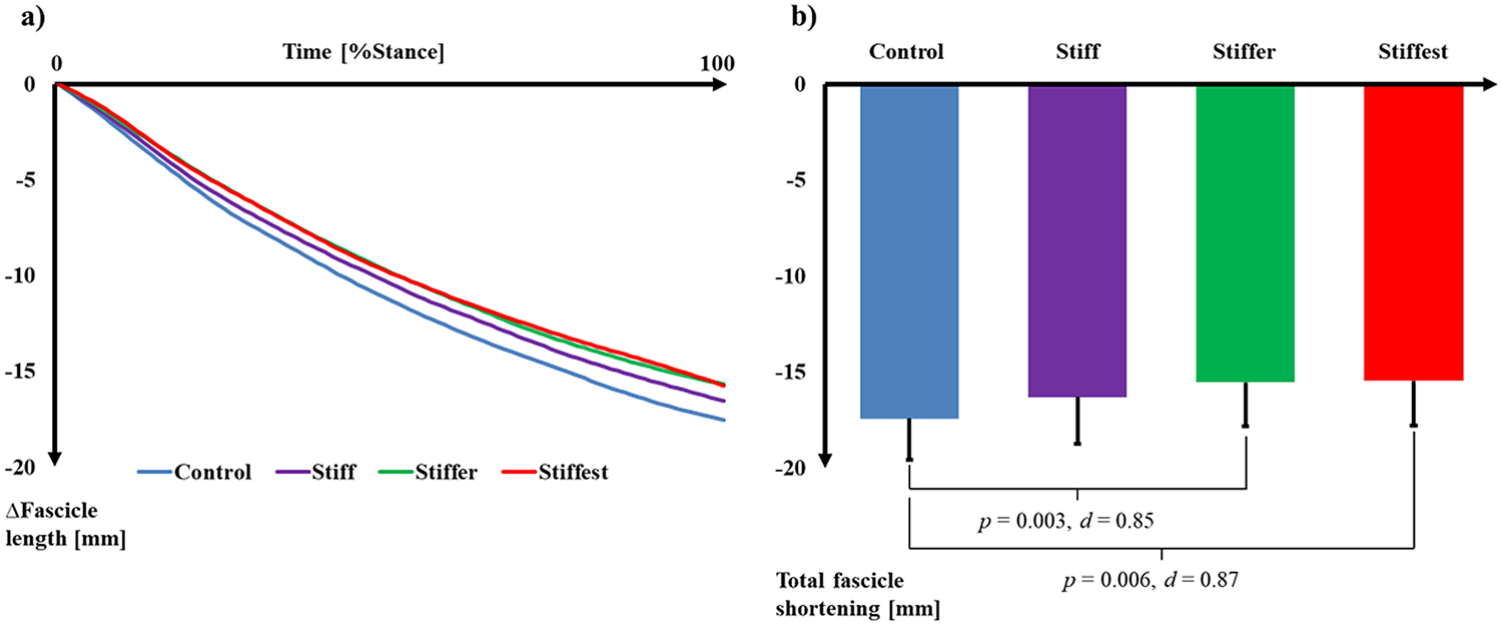
(**a**) Time-normalised, average (n = 14) change in gastrocnemius medialis muscle fascicle length over the stance phase of running relative to the length at heel-strike. (**b**) Average (± SD; n = 14) total gastrocnemius medialis fascicle shortening over the stance phase of running. *p* values and Cohen’s *d* effect sizes denote significant (*p* ≤ 0.017) differences between conditions.

**Figure 2:**
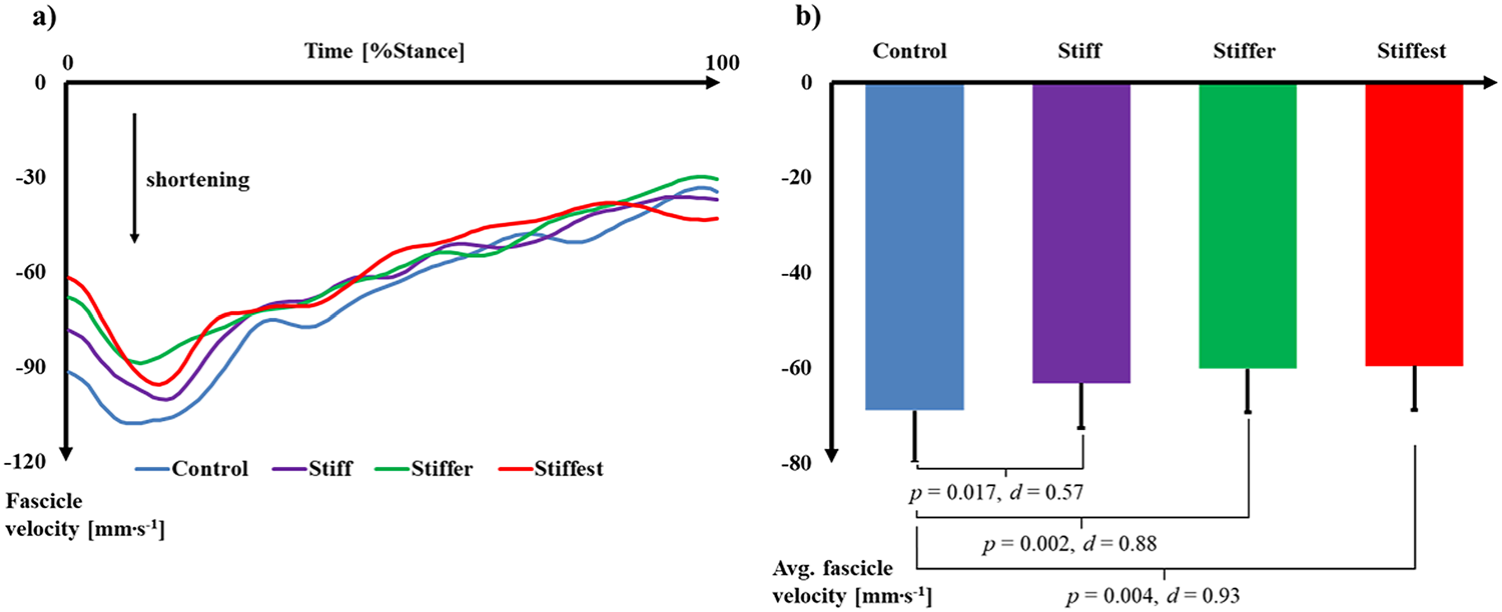
(**a**) Time-normalised, average (n = 14) gastrocnemius medialis muscle fascicle velocity over the stance phase of running. (**b**) Average (± SD; n = 14) gastrocnemius medialis fascicle velocity over the stance phase of running. p values and Cohen’s *d* effect sizes denote significant (*p* ≤ 0.017) differences between conditions.

Significant effects of MBS were observed on GM fascicle length at toe-off (*p* = 0.020), total change of GM fascicle length (*p* = 0.001), and average fascicle velocity (*p* = 0.010) during stance. There was no effect of MBS on fascicle length at heel-strike (*p* = 0.223). After correcting for multiple comparisons (*p* ≤ 0.017), GM fascicle length at toe-off in Stiff (*p* = 0.024, *d* = 0.21), Stiffer (*p* = 0.021, *d* = 0.22), and Stiffest (*p* = 0.021, *d* = 0.35) was not different compared to Control (Table 1). Pairwise comparisons revealed significant differences in total change of fascicle length between Control and Stiffer (*p* = 0.003, *d* = 0.85) and Stiffest (*p* = 0.006, *d* = 0.87) (Fig. 1). There were also significant differences in average fascicle velocity between Control and Stiff (*p* = 0.017, *d* = 0.57), Stiffer (*p* = 0.002, *d* = 0.88), and Stiffest (*p* = 0.004, *d* = 0.93) (Fig. 2).

**Table 1:** Measured dynamics and modelled energetics (mean ± SD; n = 14) for the gastrocnemius medialis (GM) muscle and Achilles tendon (AT).

Variable	Control	Stiff	Stiffer	Stiffest
E_run_ (J kg^−1^ m^−1^)	4.85 ± 0.31	4.85 ± 0.37	4.75 ± 0.36	4.85 ± 0.29
GM fascicle length at heel-strike (mm)	52.0 ± 5.0	51.9 ± 5.7	51.2 ± 5.4	51.7 ± 5.4
GM fascicle length at toe-off (mm)	34.6 ± 4.9	35.6 ± 4.7	35.7 ± 4.9	36.2 ± 4.7
GM total length change (mm)	− 17.4 ± 2.1	− 16.3 ± 2.4	− 15.5 ± 2.3*	− 15.5 ± 2.3*
Average GM shortening velocity (mm s^−1^)	− 69.0 ± 10.9	− 63.2 ± 9.4*	− 60.1 ± 9.2*	− 59.6 ± 9.2*
GM efficiency (%)	35.5 ± 4.0	35.2 ± 4.7	35.8 ± 4.5	35.2 ± 4.3
GM energy cost (J kg^−1^)	0.237 ± 0.043	0.226 ± 0.047	0.231 ± 0.057	0.222 ± 0.054
GM fascicle work (J kg^−1^)	0.084 ± 0.015	0.078 ± 0.013	0.082 ± 0.017	0.077 ± 0.017
Peak GM force (N kg^−1^)	11.63 ± 1.40	11.73 ± 1.29	11.94 ± 1.51	11.81 ± 1.48
GM force per crossbridge (pN)	2.55 ± 0.07	2.59 ± 0.05	2.60 ± 0.05*	2.61 ± 0.05*
In-series sarcomeres (count in 10^3^)	30.521 ± 3.490	31.193 ± 3.812	31.019 ± 3.841	31.525 ± 3.406
Cycles per crossbridge (count)	46.01 ± 6.10	43.15 ± 5.17	41.60 ± 5.59*	41.22 ± 5.30*
AT energy return (J kg^−1^)	0.15 ± 0.03	0.17 ± 0.04*	0.18 ± 0.04*	0.19 ± 0.05*
AT force (N kg^−1^)	58.96 ± 6.69	59.76 ± 6.47	60.92 ± 7.68	60.38 ± 7.60

*Significant (*p*  ≤ 0.017) differences compared to Control.

There was no significant difference between MBS conditions for GM muscle energy cost (*p* = 0.469). With regards to the estimated GM energy cost during stance, significant effects of MBS were found for estimated average force per crossbridge (*p* ≤ 0.001), the number of in-series sarcomeres (*p* = 0.013), and the number of cycles per crossbridge (*p* = 0.026). Compared to Control, estimated GM force per crossbridge was significantly higher in Stiff (*p* = 0.010, *d* = 0.59), Stiffer (*p* = 0.002, *d* = 0.83), and Stiffest (*p* = 0.001, *d* = 0.96). After correcting for multiple comparisons, the number of in-series sarcomeres did not differ between MBS conditions. The estimated number of crossbridge cycles during stance was significantly lower in Stiffer (*p* = 0.005 *d* = 0.75) and Stiffest (*p* = 0.013, *d* = 0.84). Achilles tendon energy return was also significantly different between MBS conditions (*p* ≤ 0.001), where compared to Control more energy was returned in Stiff (*p* = 0.006, *d* = 0.64), Stiffer (*p* ≤ 0.001, *d* = 0.96), and Stiffest (*p* ≤ 0.001, *d* = 1.17). No effects of MBS on peak GM force (*p* = 0.333), GM fascicle work (*p* = 0.139) or average AT force (*p* = 0.143) were observed. Combining the results for GM energy cost and fascicle work, no significant difference (*p* = 0.184) in GM efficiency was found (Table 1).

**Figure 3:**
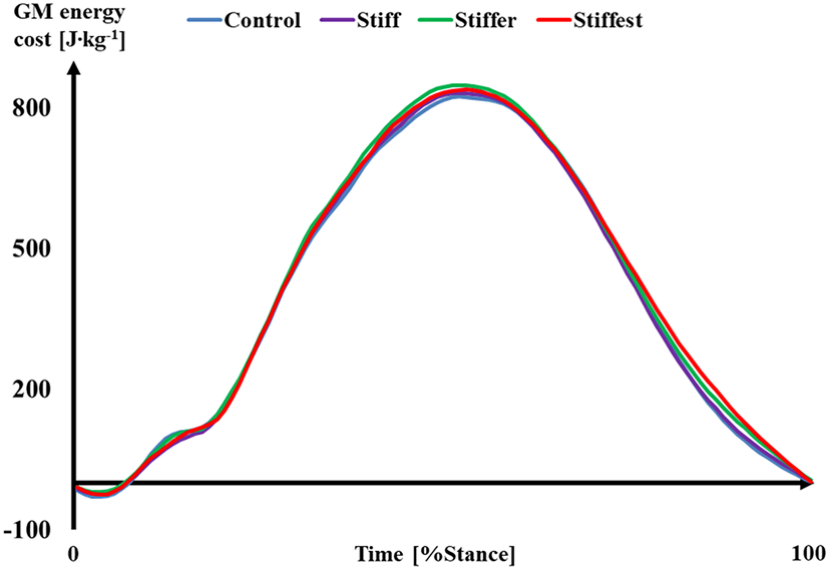
Average (n = 14) gastrocnemius medialis (GM) muscle energy cost over the stance phase of running.

There was no significant MBS × %Stance interaction (*p* = 0.946); however, a significant main effect of both MBS condition (*p* = 0.046) and %Stance (*p* ≤ 0.001) was found, suggesting that instantaneous GM muscle energy cost differed significantly across the entire stance phase (Fig. 3). There was a significant difference in instantaneous GM energy cost between MBS conditions during 0–10%Stance (*p* = 0.003) and 80–90%Stance (*p* = 0.050).

### Control vs. Best

When Control was compared to the stiff condition where E_run_ was lowest (i.e., Best), E_run_ was significantly (*p* = 0.04, *d* = 0.50) improved by 1.97 ± 1.81% (range: -0.79% to 4.71%) in Best. Compared to Control, running in Best resulted in reduced total GM fascicle shortening (Control: − 17.43 ± 2.14 mm, Best: − 15.84 ± 2.18 mm, *p* = 0.007, *d* = 0.73), slower average fascicle velocity (Control: − 68.99 ± 10.94 mm s^−1^, Best: − 61.11 ± 8.95 mm s^−1^, *p* = 0.005, *d* = 0.79), greater AT energy return (Control: 0.15 ± 0.03 J kg^−1^, Best: 0.18 ± 0.04 J  kg^−1^, *p* ≤ 0.001, *d* = 0.96), and increased average AT force (Control: 58.96 ± 6.69 N kg^−1^, Best: 60.69 ± 6.44 N kg^−1^, *p* = 0.035, *d* = 0.26). Between Control and Best, there were no significant differences in GM energy cost (Control: 0.24 ± 0.04 J kg^−1^, Best: 0.24 ± 0.06 J kg^−1^, *p* = 0.942, *d* = 0.02) or GM fascicle work (Control: 0.08 ± 0.02 J kg^−1^, Best: 0.08 ± 0.02 J kg^−1^, *p* = 0.928, *d* = 0.003).

### Lower limb kinematics and kinetics

One-way repeated measures ANOVAs and Friedman’s tests revealed significant effects of MBS on stance time (*p* ≤ 0.001); positive (*p* ≤ 0.001) and negative (*p* = 0.009) MTP, positive (*p* = 0.009) and negative (*p* = 0.001) ankle, and positive (*p* = 0.023) and negative (*p* = 0.013) hip joint work; and maximum MTP (*p* ≤ 0.001) and ankle (*p* = 0.021) plantarflexion velocity. Compared to Control, stance times were significantly longer in Stiff (*p* ≤ 0.001, *d* = 0.20), Stiffer (*p* = 0.008, *d* = 0.21), and Stiffest (*p* ≤ 0.001, *d* = 0.31); positive MTP joint work was greater in Stiff (*p* = 0.017, *d* = 0.37), Stiffer (*p* = 0.001, *d* = 0.88), and Stiffest (*p* ≤ 0.001, *d* = 0.94); positive ankle joint work was greater in Stiff (*p* = 0.008, *d* = 0.42) and Stiffer (*p* ≤ 0.001, *d* = 0.47); positive knee joint work was lower in Stiffest (*p* = 0.015, *d* = 0.33); positive hip joint work was greater in Stiffest (*p* = 0.009, *d* = 0.30); negative MTP joint work was lower in Stiffest (*p* = 0.017, *d* = 0.38); negative ankle joint work was greater in Stiffer (*p* = 0.004, *d* = 0.39); negative hip joint work was greater in Stiffest (*p* = 0.008, *d* = 0.32); and maximum MTP (*p* ≤ 0.001, *d* = 1.24) and ankle (*p* = 0.017, *d* = 0.49) plantarflexion velocities were lower in Stiffest. There was no effect of MBS (*p* = 0.462) on total lower limb joint work (i.e., sum of positive and negative work across all joints) (Supplementary Table S5).

## Discussion

The primary purpose of this study was to investigate if the total shortening and the average shortening velocity of the GM muscle is reduced when running in shoes with increased MBS. The hypothesis that total GM fascicle shortening would not be affected by MBS was rejected as running in Stiffer and Stiffest significantly reduced the amount of fascicle shortening compared to Control. The second hypothesis that running in footwear with increased MBS would reduce average GM shortening velocities was accepted. This study provides the first direct evidence that muscle function can be altered by increasing the MBS of running shoes. In fact, the lower GM shortening velocities, in theory, should reduce contractile energy cost as a result of the muscle’s force–velocity relationship^24^; however, this hypothesis could not be supported here since no significant reductions in the estimated GM muscle energy cost were shown. Although there was a substantial reduction (~ 5%) in estimated GM energy cost when running in Stiffest compared to Control, the differences shown were not significant and of small to medium effect across all conditions. Contrary to our null hypothesis that AT energy return would not be affected by MBS, AT energy return, in fact, was significantly greater in all stiff conditions compared to Control. While GM energy cost was lower in the stiff conditions (Table 1) compared to Control, this difference was not significant. Considerable between-subject variability likely precluded a significant MBS effect on estimated GM energy cost.

Although the GM fascicles shortened less with increased MBS, the sagittal ankle joint angles did not differ between conditions (Supplementary Fig. S4). Therefore, it is likely that the AT performed the outstanding “shortening” of the entire shank MTU resulting in more energy returned. This increased AT energy return when running in stiffer shoes could potentially allow the ankle plantarflexor muscles to continue to operate on a more favourable position of the muscle’s force–length–velocity relationship, reducing the required level of muscle activation to achieve a target force and thus should improve E_run_^24,25^. It is important to consider the potential benefits of running in stiff shoes during runs of longer periods of time than what was measured in this study. It has previously been demonstrated that the E_run_ progressively increases during a long-distance run^26–29^. This increase was partially related to the increase in muscle contractile energy cost^30^ and/or the redistribution of joint work to MTUs surrounding more proximal MTUs (e.g., knee), which are speculated to metabolically be more costly than the triceps surae muscles and AT^31^. It has been suggested that the tendons of more proximal MTUs are less equipped for elastic energy storage and return^31^, which would require the muscle in series to perform additional work^32^. This additional muscle work would require greater active muscle volume, potentially elevating the metabolic cost of running^32^. Together, running in stiffer shoes may reduce muscle fatigue during long-distance running events, delay the onset of joint work redistribution^33^, and therefore mitigate progressive increases in E_run_ normally seen when running in less stiff footwear. Future studies should aim to investigate how in-vivo muscle and tendon dynamics change during a prolonged run in footwear with different MBS.

Based on the findings of Cigoja et al.^18^, it was expected to find differences in GM shortening velocities towards the end of stance. The highest GM shortening velocities, however, were observed during early stance (~ 0 to 10% of stance). During this period, the generated muscular forces were estimated to be lower compared to later instances of stance due to relatively low GRFs immediately after heel strike. It is therefore speculated that the GM muscle shortened in early stance, while the GRFs are relatively small, to stretch the AT. This “pre-tuning” of the tendon would then allow the AT to stretch even further as the ankle joint undergoes dorsiflexion, and potentially stores elastic energy within the tendon. A portion of this AT elastic energy is speculated to subsequently be returned to the runner.

Although significant effects of MBS on GM shortening and shortening velocity were observed, neither E_run_ nor GM energy cost differed significantly between stiffness conditions. It is possible that MBS not only affected GM function but also the shortening and/or shortening velocity of other lower limb muscles. Previous studies have shown that MBS can result in a redistribution of lower limb joint work, where more positive work is performed at the MTP joint and less positive work is performed at the knee joint^13^. Similarly, the findings of this study showed significantly more positive work performed at the MTP and ankle joint and less positive work performed at the knee joint when running in the stiffer conditions (Supplementary Table S5). It is therefore plausible to speculate that not only the function of distal MTUs are affected by MBS but also the MTUs surrounding more proximal joints (e.g., the knee and hip). The MBS may have affected different MTUs in such a manner that “positive” and “negative” effects cancelled each other out so that no differences in whole-body E_run_ were observed during a 5 min. and 30 s-run. Future studies should attempt to synchronously record muscle fascicle and tendon images of multiple MTUs to investigate if different MTUs are affected differently by the MBS of shoes.

A recently published article investigated the effects of running with differently stiff carbon fibre plates on soleus muscle fascicle dynamics and running economy^34^. The authors found no systematic differences in soleus fascicle pennation, force, length, velocity, or stride-average soleus active muscle volume between stiffness conditions. Beck et al.^34^ therefore concluded that running with carbon fibre plates may not improve running economy and long-distance running performance. There were some methodological differences between Beck et al.’s study and ours that need to be considered when comparing the findings. First, the muscle under investigation differed between studies. Although it has been shown that the three triceps surae muscles function similarly during stance (i.e., they consistently shorten throughout ground contact) across a range of running speeds^35^, Lai et al.^35^ demonstrated that there can still be differences in fascicle length changes between the soleus and GM muscle. More specifically, the GM muscle shortens to a greater extent than the soleus muscle during running. It is therefore reasonable to speculate that if differences in muscle fascicle shortening and shortening velocity exist when running in various stiffness conditions, then these differences will likely be found at a muscle that undergoes a greater range of length changes. Second, there are considerable morphological differences between the GM and soleus muscle. For example, the GM muscle contains a higher percentage of Type II (or fast-twitch) fibres than the soleus^36^. The fact that the GM muscle predominantly consists of fast-twitch fibres allows it to generate greater forces during locomotion compared to the soleus^37^. In-vivo measurements of the GM and soleus forces in an animal model have shown that GM forces increase by a factor of 5 when transitioning from a walking to a running speed^37^. Peak soleus force increases, however, were negligible. The effects of high running speeds on GM and soleus forces become evidently important when considering that participants in Beck et al.’s study^34^ were running at a constant speed of 3.5 m s^−1^; whereas, our participants ran at 90% of individual sLT, which was for only 3 out of 17 participants below 3.5 m s^−1^.

Upon their release, the Vaporfly shoes varied in many footwear features from conventional running shoes: curved carbon fibre plate, resilient midsole material, high stack height (i.e., midsole thickness), etc. This current study cannot explain how each of these features affected lower limb muscle function and consequently E_run_ during the most recent world record runs. This study can only address how systematically increasing the MBS of shoes affects GM muscle and AT tendon energetics in running. When Control was compared to Best, a ~ 2% improvement in E_run_ was observed on average when running in shoes with straight carbon fibre plates. Previous studies reported improvements in E_run_ of 4% on average when running in the Vaporfly shoes^1,3–5^. We speculate that similar reductions in total GM shortening and shortening velocity could be observed in Vaporfly compared to ‘conventional’ running footwear. This could potentially explain parts of the 4% metabolic energy savings when running in Vaporfly. The fact that the metabolic savings in Vaporfly shoes were reported to be twice as high as in our study suggests that other mechanisms produced by the Vaporfly shoes contributed to additional metabolic cost savings. One possible characteristic would be the curved design of the carbon fibre plate. Nigg et al.^14^ suggested that a curved carbon fibre plate could act as a ’teeter-totter’, where a reaction force is created at the heel as the runner’s force is applied at the forefoot and the centre of pressure moves anteriorly, passed the pivot point of the plate. This ’teeter-totter’ mechanism was speculated to be one of the main contributors of improved E_run_ when running in the Vaporfly shoes and could account for some of the outstanding 2% in metabolic savings of the current study. Although curved plates were speculated to have a different function than the straight plates, the biomechanical findings in this study (e.g., stance times and ankle and MTP joint work, angular velocities, and angles) were comparable to previously reported result when using curved plates^1–3^. Therefore, it is plausible to speculate that the GM muscle was similarly affected during the recent world record runs. Future studies, however, should investigate how the curvature of carbon fibre plates affects muscle and tendon dynamics during running.

In our study, there were no group effects of MBS on E_run_. This is in accordance with some studies^34,38,39^ but contrary to other studies^11,22^. More importantly, however, when Control was compared to Best (i.e., plated condition, which resulted in lowest E_run_), an average improvement in E_run_ of 1.97% was observed in the best plated shoe condition. This is in accordance with the findings by Madden et al.^38^. The authors, at first, demonstrated that there were no differences in running economy between a control and stiff shoe; however, once they split up their runners into responders and non-responders, the responders showed an average improvement in running economy of 2.9% when running in the stiff shoe condition. A 2.9% improvement in running economy would correspond to a performance improvement of 1.93%^40^ or 2.26%^41^, which on a 2-h. marathon would correspond to a decrease in running time of ~ 139 or ~ 163 s. Stefanyshyn and Fusco^10^ showed group improvements in sprinting performance (i.e., in absolute time) of ~ 0.47% when the stiffness of sprinting spikes was increased by inserting straight carbon fibre plates with a stiffness of 47 N·mm^−1^ in athletes’ own footwear. More importantly, however, the athletes had their individual best performance in different stiffness conditions. When these subject-specific performance responses were taken into account, mean improvements in the best stiffness condition of 1.2% were observed compared to the own shoes. This not only demonstrates that subject-specific performances responses exist when the stiffness of shoes is increased, but also that performance improvements are much greater (i.e., 2.5-fold) when the individual effects are taken into account compared to group differences. Additionally, it shows that although there may^11,22^ or may not^34,38,39^ be significant group differences (e.g., Control vs. Stiff vs. Stiffer vs. Stiffest), the true effect of MBS on performance is greatly underestimated if the subject-specific responses are not taken into account. In our study, an average improvement in E_run_ of 1.97% in Best would correspond to ~ 94^40^ or ~ 111^41^ s on a 2-h. marathon. It needs to be acknowledged, however, that a comparison between Control and one of three plated conditions (i.e., Best) increases the probability of obtaining a false positive finding.

There are some limitations and assumptions associated with this study. We used a single GM muscle fascicle as a representation of the entire triceps surae muscle group. It is possible that regional differences in muscle fascicle properties (i.e., distal vs. proximal end of the muscle) exist^42^. The imaged area of the GM muscle, however, was consistent between footwear conditions within a subject, and therefore it is assumed that regional differences in fascicle properties only minimally affected the interpretation of the results of this study. Further, the force acting through the AT was assumed to be solely generated by the GM muscle. This is not true as other muscles (e.g., gastrocnemius lateralis, soleus, etc.) also contribute to ankle joint plantarflexion. Therefore, the absolute GM force, fascicle work, and energy cost were likely overestimated. In a study of a within-subject design, however, this assumption would not affect the interpretation of the results. Further, it needs to be noted that the AT length changes were not directly assessed during the stance phase, as direct measurements of tendon length changes during running are challenging, and do not fit theoretical expectations^43^. We calculated tendon length changes from GM muscle fascicle and MTU length changes taking the ankle and knee joint angles into account^44^. This assumes that the aponeurosis is of equal stiffness as the free tendon, and does not contribute to the storage and return of elastic energy, which may not be true^45,46^, and could change with the level of muscle activation^47^. Thus, we have likely underestimated the total amount of energy return by the AT (and aponeurosis), particularly in instances where AT force was high. Lastly, in order to aid in the interpretation of changes in E_run_ between Control and Best, the smallest worthwhile change (SWC) as measured by the metabolic cart was determined. The SWC for E_run_ was calculated to be 0.95%. It needs to be noted that not all participants exhibited changes in E_run_ greater than the SWC. For 9 out of 14 participants, however, the change in E_run_ between Control and Best was greater than 0.95% (range 1.00–4.71%).

## Conclusions

This study is the first to provide direct evidence that gastrocnemius medialis muscle function can be altered when running in shoes with increased midsole bending stiffness. Specifically, when the stiffness of running shoes is increased ‘optimally’, the gastrocnemius medialis shortening velocities were reduced and Achilles tendon energy return was increased. These effects on gastrocnemius medialis muscle and Achilles tendon function could be one of the underlying mechanisms of increased performance when running in shoes with embedded carbon fibre plates.

## Materials and methods

### Participants

Seventeen trained male runners (mean ± SD; age: 26.6 ± 5.9 years, height: 1.78 ± 0.08 m, mass: 70.7 ± 9.1 kg) visited the laboratory on two separate occasions. Participants were included in this study if they were able to run 10 km in under 44 min., were free from lower limb injuries 6 months prior to testing, and fit a size 9, 10, or 11 US running shoe. This study was approved by the University of Calgary Conjoint Health Research Ethics Board (REB19-1432). All experiments were performed according to the approved protocol and in accordance with the ethical standards of the Declaration of Helsinki. All participants gave written informed consent prior to participating in this study. Where relevant, informed consent was obtained from participants for publication of identifying information/images in an online open-access publication.

### Footwear conditions

The control condition (Control) consisted of a Nike Free Run 2018 (Nike Inc., Beaverton, USA) shoe. The stiff conditions were achieved by inserting straight carbon fibre plates of 1 mm (Stiff), 1.5 mm (Stiffer), and 2 mm (Stiffest) thickness between the factory insole and midsole, and along the full length of the shoe. The MBS of the shoes was determined by a 3-point bending test^11,13^, where the forefoot of the shoe was placed on a custom-made structure with two supporting pins. An indenter was attached to an ElectroPuls E10000 Linear-Torsion testing machine (Instron, Norwood, USA), which applied a compressive force vertically in the area of the MTP joint. The testing machine was set to displace the shoe by 20 mm at a speed of 10 mm s^−1^. This was repeated ten times for each shoe size and condition. The force and displacement data were filtered using a dual pass 2nd order Butterworth filter with a cut-off frequency of 5 Hz. The slope of the force–displacement curve (i.e., stiffness) was determined for all ten loading cycles. The stiffness values were first averaged between 80 and 90% of each loading curve (i.e., linear portion of the force–displacement curve) and then across all ten cycles. The shoes’ hysteresis (i.e., lost energy) was determined as the difference in mechanical energy that was stored during the loading phase and that was returned during the unloading phase. The lost energy was represented as a percentage of the energy stored during the loading phase. The MBS and hysteresis values were: Control, 1.58 N mm^−1^ and 35.26%; Stiff, 4.02 N mm^−1^ and 22.72%; Stiffer, 6.46 N mm^−1^ and 23.91%; Stiffest, 13.28 N mm^−1^ and 22.37%. The footwear conditions were weight matched by inserting small masses in the forefoot, midfoot, and rearfoot region of the control shoe. Across all conditions, the masses were 259, 280, and 296 g for the size 9, 10, and 11 US shoes, respectively.

### Data collection

On the first visit, individual speed at lactate threshold (sLT) was determined based on methods described elsewhere^48^ while participants ran in Control on a treadmill (Bertec Corporation, Columbus, USA) with no gradient. In brief, participants first performed a 5-min. warm-up at self-selected speed. Immediately after the warm-up, a fingertip blood sample was taken to determine resting blood lactate concentration (Lactate Pro, Sports Resource Group Inc., Minneapolis, USA). After the warm-up, the treadmill belt speed was increased by 0.8 km h^−1^ every two minutes, after which blood lactate concentration was measured. This was repeated until blood lactate concentration rose more than 1 mmol from the previous sample. sLT was determined as the speed at the stage preceding the final stage.

On the second visit, participants ran for 5 min. and 30 s at 90% of sLT in each shoe condition, respectively. The order of conditions was randomised, and participants rested for 15 min. between footwear conditions to minimise potential fatigue effects. Subjects were asked to refrain from extensive physical activity for 24 h. before the testing session. Prior to running in each footwear condition, participants ran at 3 m s^−1^ for 3 min. to get accustomed to running in the shoe conditions and with the measurement equipment. Immediately after the 3-min. familiarisation run, speed was increased to 90% of individual sLT for 5 min. and 30 s. Gastrocnemius medialis (GM) muscle fascicle images of the right leg were acquired using B-mode ultrasound imaging and a flat-shaped, 60 mm-long, linear probe (LV8-5N60-A2, ArtUs, Telemed, Vilnius, Lithuania) with a centre frequency of 8 MHz and a sampling frequency of 154 Hz. The GM muscle was chosen over other triceps surae muscles because Lai et al.^35^ showed that muscle fascicle length changes during the stance phase of running were greatest at the GM muscle. Indirect calorimetry was used to measure breath-by-breath rate of oxygen uptake (${\dot{\text{V}}}_{{{\text{O}}_{{2}} }}$ in ml kg^−1^ min^−1^) and carbon dioxide output (${\dot{\text{V}}}_{{{\text{CO}}_{{2}} }}$ in ml kg^−1^ min^−1^) using a metabolic cart (Quark CPET, COSMED srl, Rome, ITA). Prior to each testing session, the metabolic cart was calibrated using room air and a gas mixture of known composition. The flow sensor was calibrated manually with a 3-l syringe. Three-dimensional kinematic and kinetic data of the right lower limb were recorded at 200 and 1000 Hz using eight high-speed video cameras (Vicon Motion Systems Ltd., Oxford, UK) and a force-instrumented treadmill (Bertec Corporation, Columbus, USA), respectively. Retroreflective markers of 12 mm diameter were mounted on anatomical landmarks of the pelvis and right lower limb according to Cigoja et al.^13^. Kinematic, kinetic, and ultrasound data were synchronously recorded for approximately 20 s starting at the end of minute 5 of each run, which resulted in 28–33 steps of the right lower limb per footwear condition and participant, respectively. The middle 25 steps were identified and used for further analyses. Breath-by-breath metabolic data were recorded for the entire duration of the run.

### Data processing and analysis

#### Ultrasound data

**Figure 4:**
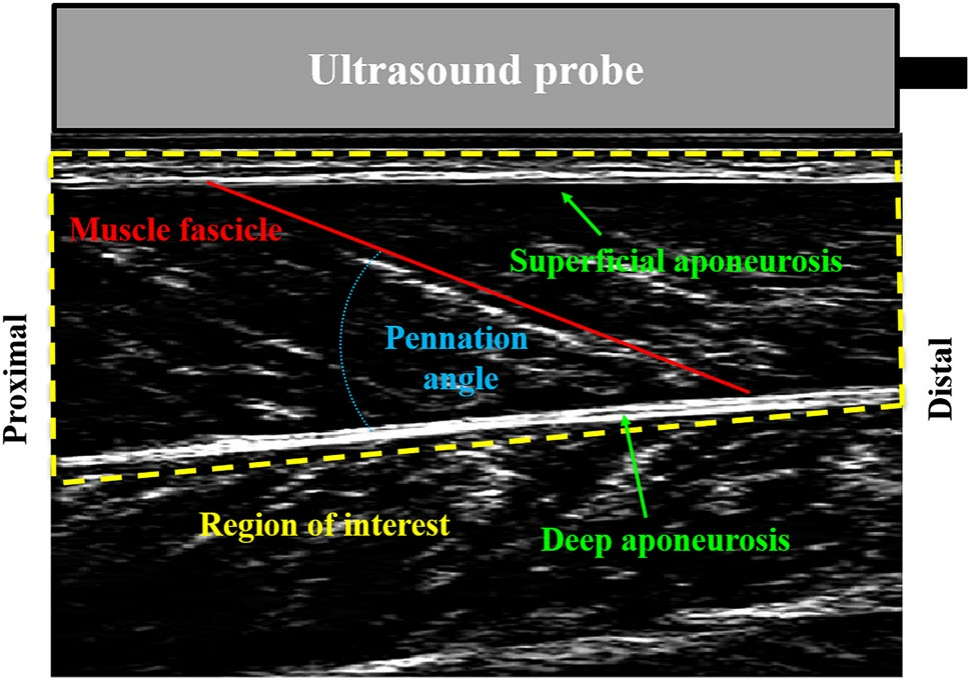
Exemplary image of the gastrocnemius medialis muscle fascicle during running.

The GM muscle fascicle images (Fig. 4) of the right lower limb were tracked using a semi-automated tracking algorithm^49^. Manual adjustments were made when the tracking algorithm did not identify the fascicle end points well. Muscle fascicle lengths were smoothened using a dual-pass second order (i.e., zero-lag fourth order) Butterworth filter with a low-pass cut-off frequency of 25 Hz. Muscle fascicle velocity was calculated by deriving fascicle length with respect to time. Fascicle lengths at heel-strike and toe-off, total change of fascicle length and average fascicle velocity over the stance phase of running were identified and used for further comparisons. The ultrasound data for three participants were excluded from any analyses due to insufficient quality of muscle fascicle images.

#### Gastrocnemius medialis and Achilles tendon energetics

Mechanical work performed by the GM fascicles was calculated as the integral of fascicle force and fascicle length change over the entire stance phase. Achilles tendon force was estimated based on a previously described musculoskeletal model^18,50,51^. For this, the sagittal plane ankle joint moment was divided by the AT moment arm. The AT moment arm was estimated by measuring the horizontal distances between the medial and lateral malleoli to the posterior aspect of the calcaneus using digital callipers. The medial and lateral distances were then averaged. The GM force was estimated based on the relative physiological cross-sectional area of all ankle plantarflexor muscles by multiplying the AT force by 0.1746^52^. Then, the scaled AT force was divided by the cosine of the pennation angle of the GM muscle fascicle. Positive fascicle work was considered fascicle shortening; the point during which the fascicles were generating mechanical energy during the stance phase^24^.

The GM energy cost was calculated during the entire stance phase for each footwear condition according to Fletcher and MacIntosh^53^. In brief, GM energy cost was approximated from the estimated number of active in-parallel crossbridges required to generate the calculated GM force, the number of crossbridge cycles during the stance phase, and the number of half-sarcomeres in series. To estimate the number of in-parallel crossbridges, the calculated GM force was divided by the estimated force per crossbridge. Because the force per crossbridge decreases with increasing shortening velocity^54^, the force per crossbridge was scaled to sarcomere shortening velocity at physiological temperatures^54^, assuming a crossbridge force of 3 pN under near-isometric conditions^55^. Sarcomere shortening velocity relative to maximal shortening velocity was calculated from the instantaneous fascicle shortening velocity throughout the stance phase, and assuming a maximal shortening velocity of 10.6 Length_fascicle_ s^−1^. The latter was calculated from the assumed maximal shortening velocities of Type I and Type II fibres of 4.4 Length_fascicle_ s^−1^ and 16.8 Length_fascicle_ s^−1^ at physiological temperatures^56^ and assuming the GM consisted of 50% Type I fibres^57^. The number of half-sarcomeres in series was estimated as the ratio of fascicle length during stance to half-sarcomere length, assuming an optimal sarcomere length during maximal activation of 2.6 µm at the short side of the plateau region of the sarcomere force–length relationship^58^. The number of crossbridge cycles was estimated from the amount of shortening within each half-sarcomere, which was estimated from the amount of GM fascicle length change during the stance phase. To accommodate this magnitude of shortening, we assumed the filaments move 10 nm with each crossbridge cycle^59^. Lastly, we assumed that one adenosine triphosphate (ATP) was consumed for each crossbridge cycle, and that 48 kJ were liberated per mol of ATP^60,61^. The GM energy cost was only calculated during the stance phase of running because GM force and power has been shown to be greater during stance with minimal to no force and power during swing^62^. It can therefore be assumed that the energy consumed by the GM muscle is negligible during swing compared to stance.

The shank MTU length was calculated based on methods proposed by Hawkins and Hull^63^ using the instantaneous ankle and knee joint angles. Instantaneous tendon length was estimated by subtracting the GM fascicle length from the shank MTU length, taking the effect of muscle pennation angle into account^64^. Achilles tendon power was calculated by multiplying tendon force by tendon lengthening/shortening velocity at each instant during the stance phase. Tendon work was determined by integrating AT power relative to time. Positive tendon work represented the utilization of elastic strain energy that was stored earlier in the stance phase^65^.

#### Metabolic data

The E_run_ (in J kg^−1^ m^−1^) was calculated as the breath-by-breath energy expenditure averaged across the final minute of each run, relative to body mass and distance covered within the last minute of the run for each condition, respectively. The Peronnet and Massicotte equation^66,67^ was used to convert the measured O_2_ consumption and CO_2_ production to units of energy (Joule). The best stiff condition was determined as the condition with a carbon fibre plate, which resulted in the lowest E_run_. To aid in interpretation of changes in E_run_, we calculated the technical error of measurement using the root mean square method^68,69^. The technical error (TE) of measurement was determined using VO_2_ averaged across four intervals of 15 s during the final minute of running in the control condition. Then, the SWC, which represents the smallest difference between conditions that can be considered “meaningful”^70,71^, was calculated as 0.5 × TE^72^. The SWC in E_run_ was considered 0.95%.

#### Kinematic and kinetic data

Raw kinematic and kinetic data of the right lower limb were analysed using a custom written MATLAB code (Version R2019a, the MathWorks Inc., Natick, USA). Force data were downsampled to 200 Hz to match the motion capture data. Kinematic and kinetic data were then smoothed using a dual-pass second order Butterworth filter with a low-pass cut-off frequency of 50 Hz. Cardan angles were calculated for the MTP, ankle, knee, and hip joint with a flexion–extension, abduction–adduction, and internal–external rotation sequence. An inverse dynamics approach was performed to determine internal joint reaction moments. Instantaneous joint power was calculated as the dot product between joint moment and angular velocity for the sagittal, frontal, and transverse plane, respectively; then, joint power was summed across planes. Positive and negative joint work was calculated as the time-integral of positive and negative joint power, respectively.

#### Statistics

Values are presented as mean ± standard deviation, unless otherwise indicated. Shapiro–Wilk tests were performed to test for normality of all dependent variables. If the Shapiro–Wilk test revealed normal distribution, one-way repeated measures analysis of variance (ANOVA) was used to test effects of MBS on a dependent variable; otherwise, the non-parametric equivalent (i.e., Friedman test) was used. Where the ANOVA or Friedman test revealed significant effects (i.e., *p* ≤ 0.050) of MBS, multiple pairwise comparisons were performed using two-tailed Student’s paired *t* test or Wilcoxon’s signed-rank test to test for differences between Control and each stiff condition (i.e., Control vs. Stiff, Control vs. Stiffer, and Control vs. Stiffest). For multiple comparisons, the significance level was set to *p* ≤ 0.017 (0.050 ÷ 3 comparisons = 0.017). Effect sizes were quantified using Cohen’s *d* and were considered small, medium, and large for *d* ≥ 0.2, *d* ≥ 0.5, and *d* ≥ 0.8, respectively^73^. A two-way ANOVA (MBS × %Stance) with repeated measures (MBS) was used to test for differences in GM muscle energy cost across the entire stance phase. When there was no significant interaction but a significant main effect was found, Tukey’s post-hoc test was used to detect significant differences between the MBS conditions.

## Supplementary Information

Supplementary Information 1.

Supplementary Information 2.

Supplementary Information 3.

Supplementary Information 4.

Supplementary Information 5.

Supplementary Information 6.
