# Supplementary figures and images for: Increasing the midsole bending stiffness of shoes alters gastrocnemius medialis muscle function during running
Source: Sci Rep. 2021 Jan 12;11:749. doi: 10.1038/s41598-020-80791-3 (PMC7804138; doi:10.1038/s41598-020-80791-3)

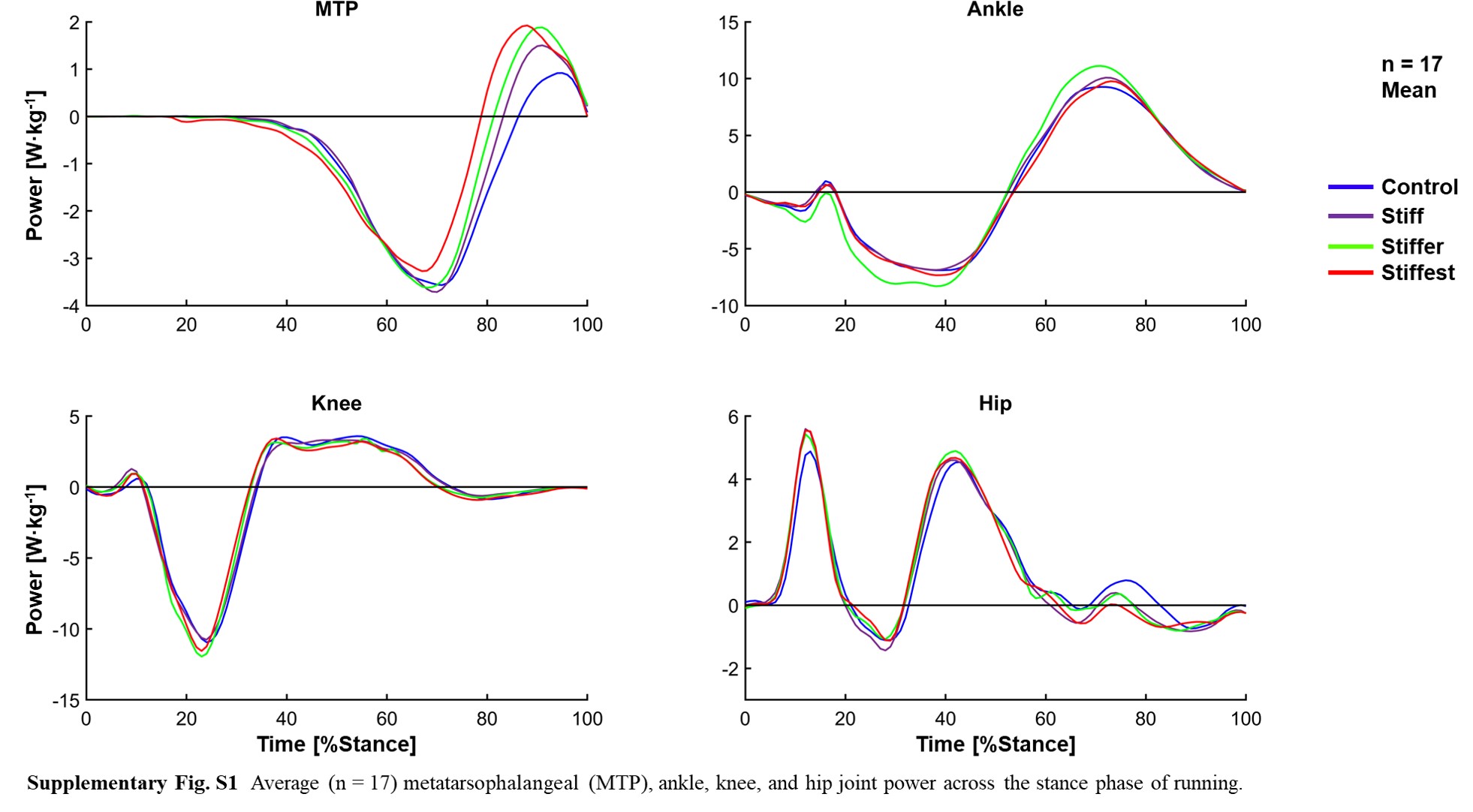

Supplement: Supplementary file 1 — Supplementary Information 1. [file 41598_2020_80791_MOESM1_ESM.jpg]

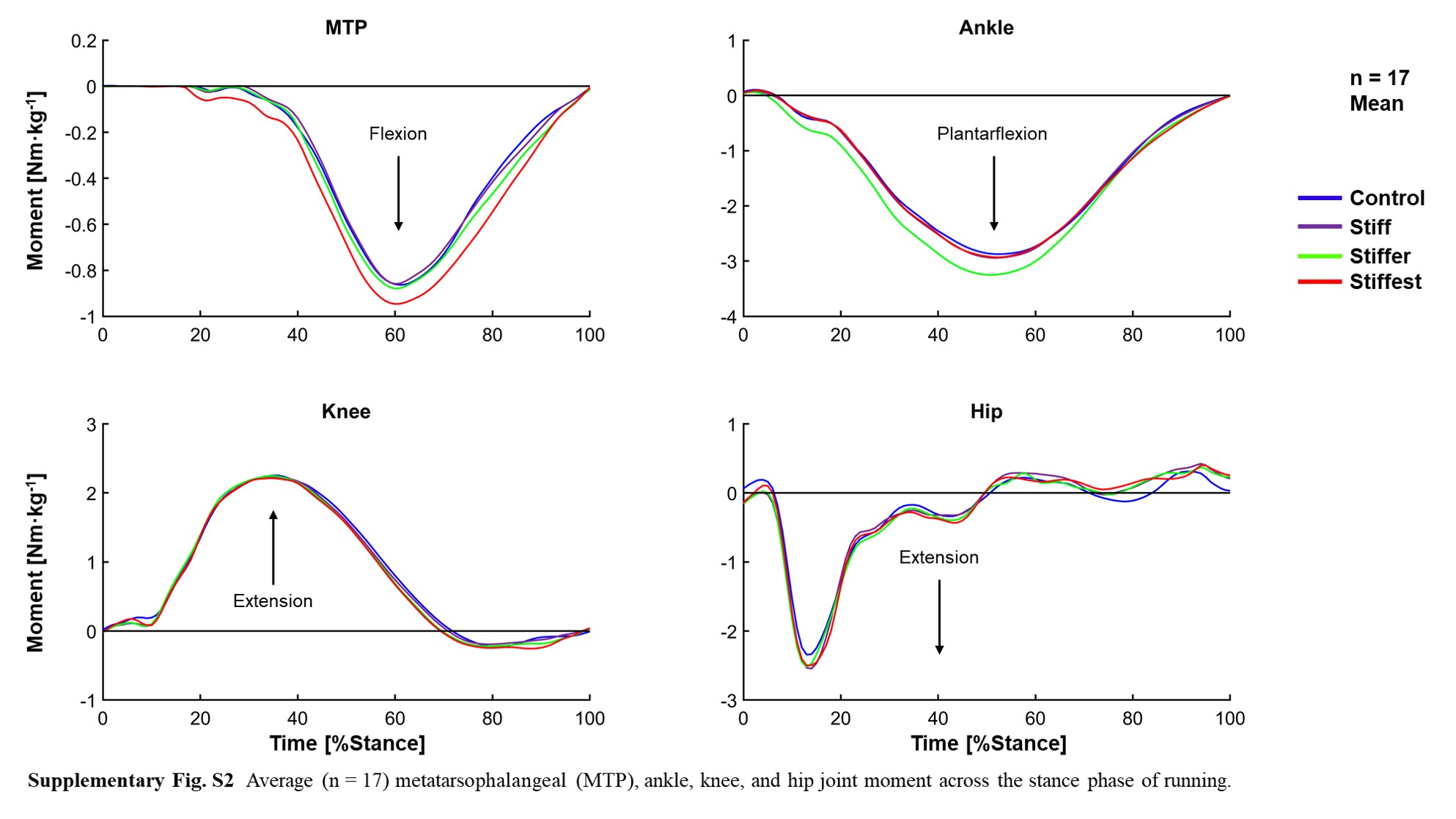

Supplement: Supplementary file 2 — Supplementary Information 2. [file 41598_2020_80791_MOESM2_ESM.jpg]

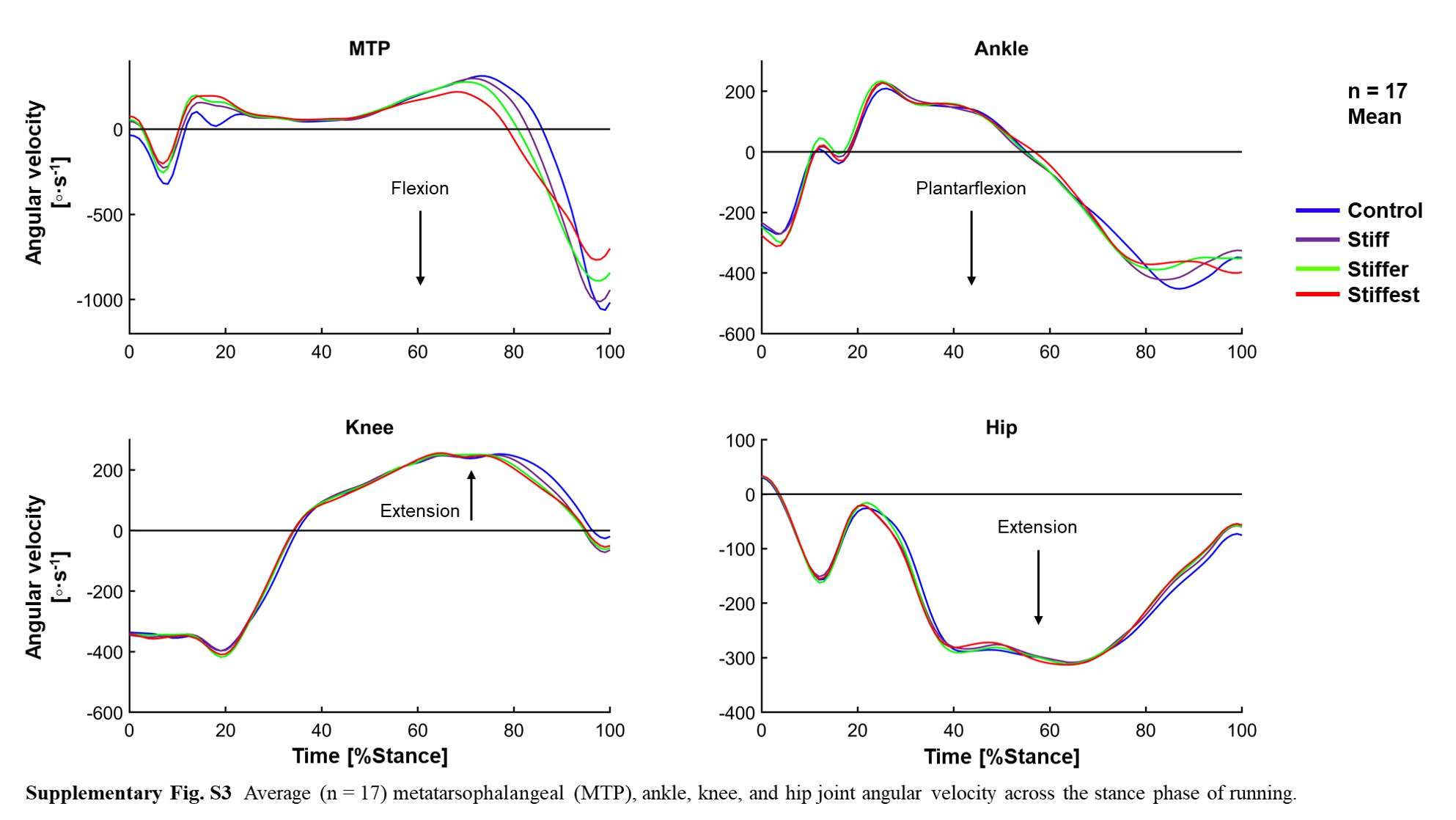

Supplement: Supplementary file 3 — Supplementary Information 3. [file 41598_2020_80791_MOESM3_ESM.jpg]

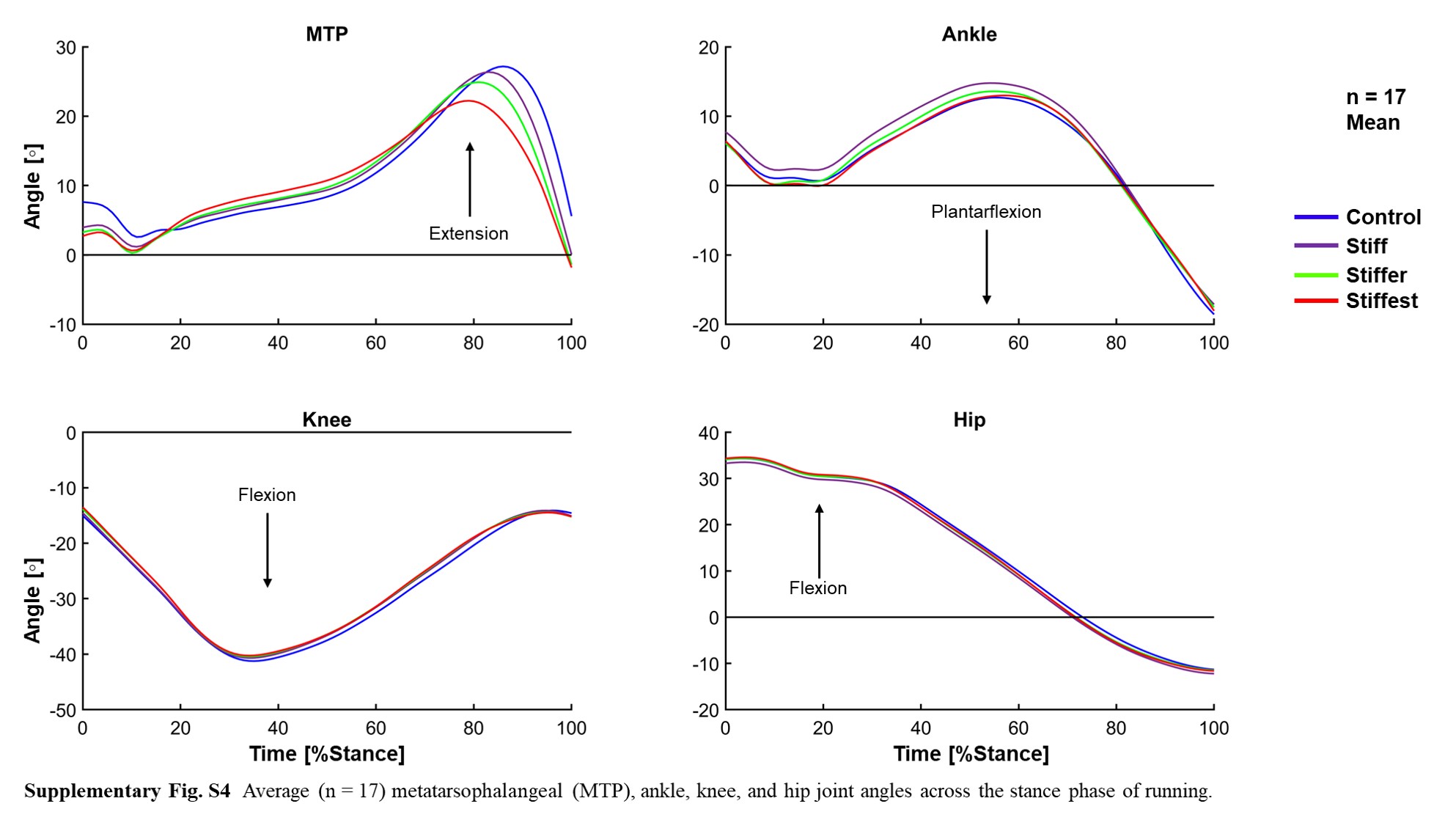

Supplement: Supplementary file 4 — Supplementary Information 4. [file 41598_2020_80791_MOESM4_ESM.jpg]
