# Supplementary material for: Increasing the midsole bending stiffness of shoes alters gastrocnemius medialis muscle function during running
Source: Sci Rep. 2021 Jan 12;11:749. doi: 10.1038/s41598-020-80791-3 (PMC7804138; doi:10.1038/s41598-020-80791-3)
Supplement: Supplementary file 5 — Supplementary Information 5. [file 41598_2020_80791_MOESM5_ESM.docx]

**Supplementary Table S5** Stance times and biomechanical descriptives (mean ± s.d.; n = 17) for the metatarsophalangeal (MTP), ankle, knee, and hip joint. * indicates significant (p ≤ 0.017) differences compared to Control.

| **Joint** | **Variable** | **Control** | **Stiff** | **Stiffer** | **Stiffest** |
| --- | --- | --- | --- | --- | --- |
| **-** | **Stance time [ms]** | 208.40 ± 19.11 | 212.12 ± 18.82* | 212.34 ± 19.09* | 214.40 ± 19.33* |
| **MTP** | **positive work [J∙kg^-1^]** | 0.07 ± 0.04 | 0.08 ± 0.05* | 0.11 ± 0.05* | 0.11 ± 0.04* |
|  | **negative work [J∙kg^-1^]** | -0.27 ± 0.06 | -0.26 ± 0.06 | -0.26 ± 0.07 | -0.23 ± 0.12* |
|  | **max. PF moment [Nm∙kg^-1^]** | -0.91 ± 0.17 | -0.90 ± 0.17 | -0.92 ± 0.16 | -1.00 ± 0.29 |
|  | **max. PF angular velocity [°∙s^-1^]** | -1064.45 ± 190.86 | -1069.11 ± 201.36 | -1013.01 ± 217.82 | -843.71 ± 165.09* |
| **Ankle** | **positive work [J∙kg^-1^]** | 0.74 ± 0.13 | 0.79 ± 0.14* | 0.86 ± 0.36* | 0.77 ± 0.15 |
|  | **negative work [J∙kg^-1^]** | -0.60 ± 0.14 | -0.61 ± 0.17 | -0.73 ± 0.46* | -0.64 ± 0.13 |
|  | **max. PF moment [Nm∙kg^-1^]** | -2.92 ± 0.34 | -2.98 ± 0.37 | -3.31 ± 1.50 | -2.97 ± 0.37 |
|  | **max. PF angular velocity [°∙s^-1^]** | -489.54 ± 83.12 | -468.64 ± 80.38 | -456.22 ± 74.24 | -451.83 ± 68.60* |
| **Knee** | **positive work [J∙kg^-1^]** | 0.52 ± 0.11 | 0.55 ± 0.17 | 0.51 ± 0.11 | 0.49 ± 0.09* |
|  | **negative work [J∙kg^-1^]** | -0.67 ± 0.16 | -0.70 ± 0.18 | -0.69 ± 0.16 | -0.67 ± 0.16 |
|  | **max. extension moment [Nm∙kg^-1^]** | 2.36 ± 0.36 | 2.39 ± 0.53 | 2.37 ± 0.37 | 2.32 ± 0.39 |
|  | **max. extension angular velocity [°∙s^-1^]** | 310.14 ± 41.59 | 311.72 ± 43.04 | 306.26 ± 43.83 | 306.93 ± 40.49 |
| **Hip** | **positive work [J∙kg^-1^]** | 0.55 ± 0.13 | 0.58 ± 0.13 | 0.59 ± 0.12 | 0.59 ± 0.15* |
|  | **negative work [J∙kg^-1^]** | -0.30 ± 0.13 | -0.35 ± 0.21 | -0.33 ± 0.14 | -0.34 ± 0.16* |
|  | **max. extension moment [Nm∙kg^-1^]** | -2.80 ± 0.59 | -2.98 ± 0.56 | -2.98 ± 0.65* | -2.94 ± 0.62* |
|  | **max. extension angular velocity [°∙s^-1^]** | -375.23 ± 29.83 | -365.13 ± 33.57 | -369.53 ± 31.48 | -371.33 ± 32.02 |
